# Selenoprotein P in a Rodent Model of Exercise; Theorizing Its Interaction with Brain Reward Dysregulation, Addictive Behavior, and Aging

**DOI:** 10.3390/jpm14050489

**Published:** 2024-05-03

**Authors:** Patrick Mohr, Colin Hanna, Aidan Powell, Samantha Penman, Kenneth Blum, Alireza Sharafshah, Kai-Uwe Lewandrowski, Rajendra D. Badgaiyan, Abdalla Bowirrat, Albert Pinhasov, Panayotis K. Thanos

**Affiliations:** 1 Behavioral Neuropharmacology and Neuroimaging Laboratory on Addictions, Clinical Research Institute on Addictions, Department of Pharmacology and Toxicology, Jacob School of Medicine and Biomedical Sciences, University at Buffalo, Buffalo, NY 14203-1014, USA; 2 Department of Molecular Biology, Adelson School of Medicine, Ariel University, Ariel 40700, Israel; 3 Division of Addiction Research & Education, Center for Sports, Exercise, and Mental Health, Western University of Health Sciences, Pomona, CA 91766, USA; 4 Cellular and Molecular Research Center, School of Medicine, Guilan University of Medical Sciences, Rasht 8813833435, Iran; 5 Department of Orthopaedics, Universitaria Sanitas, Fundación, Bogotá P.O. Box 011, Colombia; 6 Department of Psychiatry, Mt. Sinai School of Medicine, New York, NY 10029, USA; 7 Department of Psychology, University at Buffalo, Buffalo, NY 14260-4110, USA

**Keywords:** rat, aerobic exercise, selenoprotein P, SEPP1

## Abstract

Exercise promotes health and wellness, including its operation as a protective factor against a variety of psychological, neurological, and chronic diseases. Selenium and its biomarker, selenoprotein P (SEPP1), have been implicated in health, including cancer prevention, neurological function, and dopamine signaling. SEPP1 blood serum levels were compared with a one-way ANOVA between sedentary (SED), moderately exercised (MOD) [10 m/min starting at 10 min, increasing to 60 min], and high-intensity interval training (HIIT) exercised rats [30 min in intervals of 2-min followed by a 1-min break, speed progressively increased from 10 to 21 m/min]. HIIT rats showed significantly higher serum SEPP1 concentrations compared to MOD and SED. More specifically, HIIT exercise showed an 84% increase in SEPP1 levels compared to sedentary controls. MOD rats had greater serum SEPP1 concentrations compared to SED, a 33% increase. The results indicated that increased exercise intensity increases SEPP1 levels. Exercise-induced increases in SEPP1 may indicate an adaptive response to the heightened oxidative stress. Previous studies found a significant increase in dopamine D2 receptor (D2R) binding in these same rats, suggesting a potential association between SEPP1 and dopamine signaling during exercise. Modulating antioxidants like SEPP1 through personalized therapies, including exercise, has broad implications for health, disease, and addiction.

## 1. Introduction

It has been well established that exercise improves health and well-being, exerting protective effects against a variety of psychological, neurological, and chronic diseases. Among its extensive benefits, exercise has also been demonstrated to reduce cocaine-seeking behaviors in rats, showcasing its influence on the brain’s reward systems, particularly through its effects on neurotransmitters like dopamine [1,2]. Furthermore, work in our lab revealed daily high-intensity interval training (HIIT) treadmill exercise to significantly increase dopamine D2 receptor (D2R) binding in the nucleus accumbens (NAc) shell, a crucial component of the brain’s reward circuitry [3]. We also saw metabolic activation in areas involved in memory, movement, and sensory processing as a result of exercise [4]. Moderate aerobic exercise has been shown to lower inflammation and reduce depression in humans [5]. Conversely, sedentary behavior puts humans at risk for many diseases such as diabetes, cardiovascular complications, and various cancers. Overall physical activity has significantly decreased in everyday life and is considered by some to be a public health crisis [6,7].

Blood analysis studies show traces of post-exercise biomarkers that may be involved in the observed benefits of physical activity. Some good examples of this are cytokines and other inflammatory markers, which have been linked to affective symptoms [5]. In this case, higher intensity exercise produced higher levels of IL-6, a pro-inflammatory cytokine that was positively correlated with elevated levels of stress [5]. Though negative aspects of over-exercising are documented, there are many positive aspects of both high-intensity and moderate exercise. These positive aspects can be indicated in blood biomarkers as well. In the same study previously mentioned, moderate exercise decreased inflammatory biomarkers and perceived stress, and both moderate and high-intensity exercise decreased depressive symptoms [5].

One understudied biomarker with potentially significant effects is the antioxidant selenoprotein P (SEPP1). SEPP1 is an established biomarker of selenium (Se) status and has been seen to be exercise-induced [8]. In mouse plasma, SEPP1 levels were identified to be significantly upregulated due to wheel running [8,9]. It is considered a major Se-containing protein in plasma and is an important aspect of brain function [8,10]. Selenium, an integral part of selenoproteins, is needed for proper immune system functioning and looks to assist in counteracting the development of many diseases [11,12]. In mice, SEPP1 is known to act directly on the hippocampus, supporting neurogenesis and reversing symptoms of cognitive decline associated with hippocampal injury [8]. The research detailing the relationship between exercise and SEPP1 has a lot of room for development, but there have been some reports on the role of SEPP1 in neuroprotection.

Moreover, emerging research highlights the involvement of SEPP1 in dopaminergic responses to exercise. SEPP1 appears to play a role in modulating dopamine transmission, with studies suggesting its influence on dopamine release and uptake rates [13,14]. The interaction between SEPP1 and dopamine receptors, particularly D2R, may contribute to the neuroprotective effects of exercise against neurodegenerative diseases and substance abuse disorders. SEPP1 may also be involved in nigrostriatal dopaminergic transmission and neuronal survival. SEPP1 has been expressed in the nigrostriatal pathway (a brain neurocircuit essential for movement) and its antioxidant properties are believed to be protective against Parkinson’s disease (PD) [15]. Subjects with PD showed significantly decreased SEPP1 levels in postmortem brain tissue when compared with controls [15]. SEPP1 or apolipoprotein E receptor 2 (ApoER2) knockout mice also developed severe neurodegeneration in the presence of mild selenium deficiency [16]. This posits an interaction of SEPP1 and exercise against neurodegeneration as both have been shown to exert neuroprotective properties. In addition, the possible interaction both exercise and SEPP1 may have with dopamine may lead to further understanding of the intricate mechanisms underlying exercise’s beneficial effects. Exploring this relationship may offer valuable insights into novel therapeutic strategies targeting dopaminergic pathways for the management of neurodegenerative disorders and substance abuse disorders. It is well-known that dopamine and especially the D2R are involved in both focus and cognitive ability, certainly positive attributes involving well-being and functionality.

Exercise’s effects on memory and the hippocampal regions are well documented. Six weeks of moderate treadmill exercise can increase brain glucose metabolism (BGluM) in sub-hippocampal regions (Subiculum and Postsubiculum) in female rats [4]. These areas are known to respond to changes in head direction and encode for spatial orientation [17,18]. Similar sub-hippocampal increases in glucose uptake were also observed after an acute dose of cocaine in exercised rats [19]. In humans, aerobic exercise is linked to better memory and increased hippocampal volume [20,21]. The exact role of SEPP1 in this phenomenon has not yet been fully researched.

In humans, low levels of SEPP1 are strongly associated with all-cause mortality and mortality due to cancer, cardiovascular, respiratory, and gastrointestinal diseases [22,23]. Selenium deficiency may contribute to multiple neurodegenerative disorders and neurological deficits, such as Alzheimer’s disease (AD), PD, and Huntington’s disease, as well as multiple sclerosis [24]. Additionally, human genetic mutations that disrupt the synthesis of selenoproteins are associated with cerebral and cerebellar atrophy, hormonal abnormalities, and sensory deficits [25]. SEPP1 genetic variations leading to lower SEPP1 expression have been associated with an increased prostate cancer incidence [26]. To fully understand this antioxidant, its health benefits, and its therapeutic potential, more data are needed.

Currently, there are very little data involving selenoprotein levels in rats. Most rodent SEPP1 studies involve mice. This study examines the effects of differing levels of aerobic exercise on blood serum SEPP1 levels in rats. We also explore a possible correlation SEPP1 may hold with dopamine signaling. We hypothesize that exercise will influence levels of SEPP1 and that these will correlate with exercise intensity.

## 2. Materials and Methods

### 2.1. Animals

Eight-week-old male Sprague Dawley rats (*n* = 24) were individually housed in open-top cages with a 12 h reverse light/dark cycle (dark: 8:00 a.m. to 8:00 p.m.) and a set temperature of 22.0 ± 2.0 °C. The rats were acquired from Taconic, Hudson, NY. Rats were handled every day and had unrestricted access to food and water. The National Academy of Sciences Guide for the Care and Use of Laboratory Animals (1996) was followed in conducting this experiment and approval was given by the University at Buffalo Institutional Animal Care and Use Committee (RIA13095Y; 06/01/23).

### 2.2. Exercise Regimen

The rats were given a one-week habituation period to become familiar with their environment. Exercise running was conducted as previously described using a customized treadmill that was separated into six Plexiglas running lanes and encased by a piece of sheet metal [2,3]. Rats were randomly divided into a high-intensity exercise group (HIIT, *n* = 10), moderate level exercise group (MOD, *n* = 6), or sedentary control group (SED, *n* = 8). Each exercise regimen was completed between 10:00 am and 2:00 pm during the animal’s dark (active) cycle. Animals were monitored continuously throughout the exercise regimen. The MOD group began running at a constant pace of 10 m/min for 10 min per day. The workout duration was increased by 10 min every day while maintaining the same speed until a maximum rate of 60 min was attained. The animals were given a 10-min break after the first 30 min of running. Over the course of six weeks, the MOD group exercised five days a week. After the six-week regimen, the total running distance was roughly 16.5 km [27]. The HIIT group started running at 10 m/min for 30 min. The rats were subjected to a 3-min regimen consisting of two minutes of running followed by a one-minute respite. This pattern was repeated ten times for a total of thirty minutes. After five days, the speed rose to a maximum of 21.46 m/min in 2.68 m/min increments. For the remainder of the exercise program, the maximum speed of exercise was maintained. The HIIT group exercised for six consecutive weeks, 7 days a week. The SED group did not use the treadmill and instead remained in their home cages [27,28]. All rats were deeply anesthetized via isoflurane (5%), and had blood collected via cardiac puncture prior to euthanasia. Following a 30 to 45 min clotting period at room temperature, blood was spun for 15 min at 3000 RPM and 4 °C. The serum was kept at −80 °C until it was needed for analysis.

### 2.3. Selenoprotein P ELISA

SEPP1 levels were measured using ELISA approach (ELISA from LSBio, Seattle, WA, USA). The detection range of the SEPP1 ELISA was 0.156–10 ng/mL, and sensitivity was <0.094 ng/mL according to manufacturer’s instructions. Briefly, samples were diluted 1:1000 with PBS (0.02 mol/L pH 7.0–7.2). Seven standard dilutions were plated with concentrations of 10 ng/mL, 5 ng/mL, 2.5 ng/mL, 1.25 ng/mL, 0.625 ng/mL, 0.313 ng/mL, or 0.157 ng/mL. The wash stages included aspirating after adding around 350 μL of wash buffer and letting it settle for one to two minutes. Sample, standard, or blank (100 μL) were added to each well and incubated for 90 min at 37 °C. Following aspiration of the wells, 100 μL of biotinylated detection antibody was added to each well followed by incubation at 37 °C for 60 min. The wells were then aspirated and washed three times. Avidin-horseradish peroxidase (HRP) conjugate solution, 100 μL, was added to each well, then incubated for 30 min at 37 °C. Wells were aspirated and washed 5 times. TMB substrate solution (90 μL) was added, and the mixture was incubated at 37 °C for 15 min or until an appropriate color change was observed. Stop solution (50 μL) was added to each well until the solution turned yellow. Optical density (OD) was immediately read and calculated at 450 nm. All measurements were performed in duplicate. The mean concentrations derived from duplicates were used for statistical analysis.

### 2.4. Statistical Analysis

GraphPad Prism 9 was used for all statistical analysis and graphing (GraphPad Version 10 Software LLC, Boston, MA, USA). A one-way ANOVA was used to compare the plasma SEPP1 concentrations among the exercise groups. Adequacy of sample size was determined by, and is consistent with, previous publications [29]. The Anderson–Darling test for normality was conducted on all three conditions: SED (*p* = 0.577), MOD (*p* = 0.656), and HIIT (*p* = 0.095). All variables exhibited no significant departure from a normal distribution (*p* > 0.05), supporting the assumption of normality for subsequent analyses. Tukey’s multiple comparisons test was used for post hoc comparisons. A = 0.05 was set as the threshold for significance.

## 3. Results

**Figure 1:**
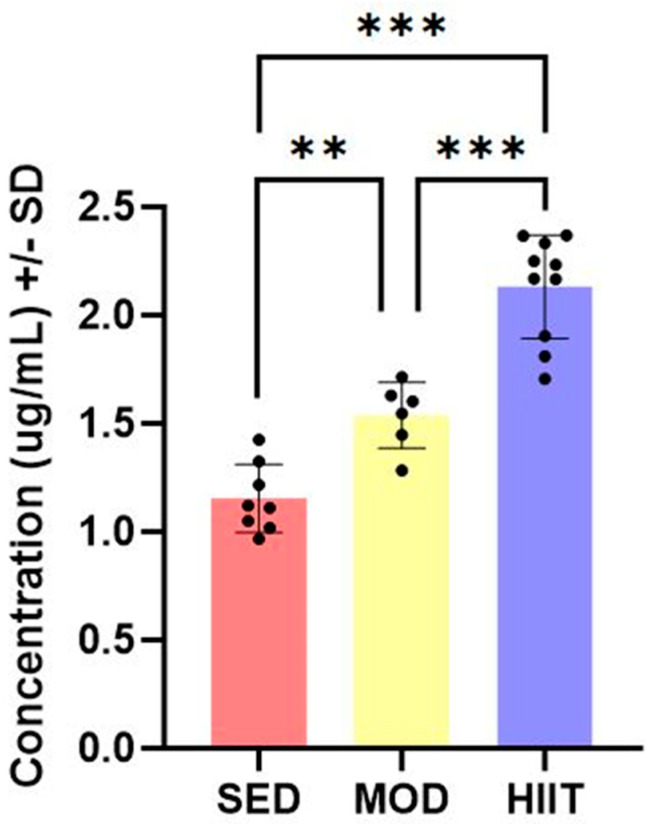
Plasma SEPP1 levels in SED (*n* = 8), MOD (*n* = 6), and HIIT-exercised (*n* = 10) rats. The data revealed a significant effect of exercise on plasma SEPP1 levels. ** *p* < 0.01, *** *p* < 0.0001.

A one-way ANOVA revealed a significant effect of exercise on plasma SEPP1 levels in SED, MOD, and HIIT rats [F(2, 21) = 56.89; *p* < 0.0001]. Pairwise comparisons showed that MOD exercise significantly increased SEPP1 levels compared to their control counterparts (SED), with an increase of 33% (*p* = 0.0043). Rats subjected to HIIT exercise exhibited an even greater increase in SEPP1 levels, with a remarkable 84% increase compared to the SED group (*p* < 0.0001). Furthermore, the HIIT group also demonstrated significantly higher SEPP1 levels compared to the MOD group (*p* < 0.0001). These results are illustrated in Figure 1.

## 4. Discussion

The findings of our study clearly demonstrated a significant increase in SEPP1 levels in male rats, directly correlating with the intensity of treadmill exercise they underwent. This intriguing observation suggests that exercise may be dose (intensity)-dependent in modulating SEPP1 expression, potentially influencing physiological functions in male rodents. SEPP1 is known to be involved in selenium transport and as a crucial antioxidant, protecting cells from oxidative damage [30,31] when there is an imbalance between the body’s capacity to detoxify reactive oxygen species (ROS) and the amount of ROS that cells and tissues produce and accumulate. Exacerbated accumulation of ROS in turn can lead to oxidative damage to DNA, RNA, proteins, and lipids, which compromise the structural integrity and function on cellular and tissue levels [32]. The exercise-induced increase in SEPP1 levels may indicate an adaptive response to the heightened oxidative stress that is associated with intense physical activity [33]. ROS accumulation is known to impact health and diseased states, especially with increasing age. From the other side, ROS generation contributes significantly to aging and the age-related decrease of muscle mass and function [34]. Beyond the physical detriments to health, oxidative stress is also linked with neurodegeneration and altered mental states. One of the main characteristics of neurodegenerative illnesses like PD and AD is the gradual degeneration of neurons. Accumulation of ROS has been observed to play a role in the onset and/or acceleration of cell death in PD and the subsequent oxidative stress is considered a key factor in the development of AD [35,36].

AD has also been linked to selenium deficiency [23]. Dietary selenium supplementation has been shown in numerous studies to have multiple beneficial effects on the alleviation of disease symptoms [37,38,39]. These include increasing neuronal activity, minimizing synaptic impairments, reducing astrocyte and microglia activation, and lowering levels of total and phosphorylated tau by inhibiting GSK-3β action [38]. Exercise is associated with enhanced antioxidant defenses, reduced inflammation, and improved synaptic plasticity, similar to the reported effects of selenium supplementation in mitigating AD-related pathology. These paralleled outcomes share results of promoting neuronal health and resilience. This hints at the possibility that exercise by itself or in combination with supplementation might offer a holistic approach to neuroprotection and potentially contribute to AD prevention or management. In addition, abnormalities in signal transduction, oxidative stress, and metal ion dyshomeostasis all contribute significantly to the onset of AD. In fact, there are a plethora of studies which indicate that selenium (Se), as Se-containing compounds or as selenoproteins, may have potential benefits in reducing the pathology associated with AD. For instance, the majority of the molecular pathways crucial to the development of AD require Se [40]. More research is needed to fully understand the neuroprotective effects mediated by Se supplementation and exercise-induced SEPP1 levels as well as potential synergistic interactions between them.

Of significance, a long-term study used exercise pretreatment to attenuate AD-related pathology. The findings revealed long-term exercise pretreatment dramatically reduced learning and memory impairment as well as anxiety- and depressive-like behaviors in a rat model of AD. In addition to the behavioral findings, pretreatment with exercise decreased amyloid-β deposition and tau hyperphosphorylation and conserved spine density, synapses, and presynaptic vesicles. Furthermore, neuronal apoptosis, degeneration, and injury were prevented by exercise. According to other studies, exercise pretreatment greatly reduces the imbalance of mitochondrial dynamics while also suppressing oxidative stress and neuroinflammation [41]. Taken together, these findings point to long-term exercise pretreatment as a potential strategy to slow neurodegenerative processes underlying different brain pathologies, including dysregulation of the brain reward system and addictive behavior.

While many studies delve into the effects of selenium supplementation on diseased states, few show how it directly affects Se status or SEPP1 levels. The available data have contrasting results. Several studies have found that Se supplementation has no significant effect on SEPP1 levels in healthy individuals [42,43]. However, SEPP1 levels have been seen to increase in those suffering from metabolic syndrome after a two-month period of selenium supplementation [43]. Additionally, another study found that Se supplementation significantly increased SEPP1 levels, reaching a plateau after two weeks with a maximum increase of 34% at 4 weeks [44]. This same study found that in a group starting with 74% higher baseline levels, supplementation had no effect. This may demonstrate that the baseline Se levels in the organism and health status could affect Se supplementation efficacy. Our experimental cohort consisted of healthy juvenile rats. Given this, we still saw an 84% increase in SEPP1 levels in the HIIT group of rats that were exposed to high-intensity exercise, suggesting that exercise may have a greater impact on SEPP1 levels than Se supplementation. Of course, a limitation in our overall interpretation might be due to differences in human compared to animal models.

The findings of this study, which indicate a positive correlation between increased levels of SEPP1 and intense exercise, offer insights into the potential development of exercise-based personalized therapeutic interventions. These interventions could serve as preventive measures against various diseases and abnormalities. Furthermore, integrating exercise into conventional therapies may synergistically enhance antioxidant effects, suggesting a promising avenue for augmentation therapy. This might be particularly relevant for individuals predisposed to conditions involving oxidative stress, such as cancer and cardiovascular and neurological diseases [45]. As previously stated, low levels of SEPP1 are associated with a heightened risk of cardiovascular issues, cerebral atrophy, hormonal abnormalities, and sensory deficits [22,25]; human compliance, using exercise as a tool to curb these risks, could have profound effects. Numerous age-related diseases affected by oxidative stress could be targets for this potential treatment. Future research into the mechanisms of this relationship could open novel pathways for personalized therapeutic interventions targeting oxidative stress-related diseases.

It was previously shown that rats exposed to physical exercises were found to have a significant increase in D2R binding compared to sedentary rats. More specifically, the HIIT rats saw an increase in D2R binding in the NAc shell (Table 1) [3], while the MOD rats demonstrated increases in D2R binding within several subregions of the dorsal and ventral striatum (Table 2) [2]. This suggests a potential relationship between SEPP1 and dopamine signaling pathways. The concurrent elevation of SEPP1 levels and D2R binding following exercise may indicate a mechanism by which SEPP1 influences dopaminergic signaling. The mechanism behind this phenomenon remains unknown but has garnered increased attention in recent research due to its implications for understanding the neurobiological effects of exercise and dopamine signaling.

This relationship is key in influencing reward deficiency and addiction. A recent study found SEPP1 modulated vesicular dopamine release in the NAc of mice mediated by D2R. They observed that the increase in dopamine release in response to methamphetamine was inhibited by the addition of exogenous SEPP1. The results suggest that SEPP1-ApoER2 binding/interaction promotes D2R activity. Possible mechanisms include direct changes in D2R function, surface expression, or crosstalk between ApoER2 and D2R intracellular signaling pathways. In the absence of SEPP1, D2R activity was decreased, allowing for the large increases in vesicular DA release seen in part of the study [14]. It is possible that exercise may have multiple mechanisms that play an important role in substance and non-substance behavioral addictions.

The D2R binding results in our previous studies are supported by similar findings among exercised rats [46,47,48,49]. Prior research has demonstrated that augmenting dopamine signaling, namely through D2R, decreases rodent drug and alcohol intake [50,51]. For example, D2R-MSN potentiation was seen to be associated with resilience towards compulsive cocaine seeking [52]. Moreover, enhancement of D2R signaling correlates with heightened motivation for addiction recovery, whereas decreased D2R levels in the striatum correlate with heightened impulsivity and addictive behavior [53]. We speculate that exercise may mitigate addictive behaviors through its compounding effects on D2R-like binding and SEPP1 expression. Additionally, exercised-induced changes in neuroplasticity and neurotransmitter regulation, such as with enhanced glutamatergic signaling (a driver of VTA-NAc dopamine release), could work in synergy with SEPP1′s effect on addiction pathways [54]. Exercise has been shown to be helpful for drug addiction of all kinds at all stages because it can reverse the molecular damage done to the dopaminergic and glutamatergic systems [55].

Our findings are novel as there are few other studies of this kind performed on rats. From our knowledge, no other study has looked at the effects of varying exercise on SEPP1 levels nor specifically high-intensity interval training. Since our study employed treadmill running rather than wheel running, the results are more relevant to exercise methods commonly used in human research and fitness programs. This approach increases the translatability of our findings to real-world applications in human health and exercise studies. A study conducted with mice investigated the impact of exercise on SEPP1 levels and found that it is induced by physical activity [8]. Additionally, hepatic SEPP1 has also been seen to be exercise-induced in mice [56]. A recent study found exercise to upregulate two other selenoproteins, selenoprotein F and selenoprotein T, in aged mice [57]. One study found moderate exercise to have no effect on circulating SEPP1 levels in normal and overweight individuals; however, this was only after one 60-min exercise session [58]. Another found regular exercise in young adults to lower plasma SEPP1 levels. Regular exercise was defined by engaging in resistance training and/or moderate-intensity treadmill activity for at least 300 min every week for a period longer than six months [59].

Unpublished research from our lab has revealed that deep in silico pharmacogenomic analyses found a strongly connected network of 13 genes demonstrating that SELENOP, SEPSECS, DIO2, DIO1, and MAPT had pharmacogenetic potential for investigating exercise-neurodegeneration-addiction networks. Variant Annotation Assessment (VAA) showed 17 structural and regulatory annotations and 38 non-structural and non-regulatory PGx variant annotations of candidate genes involved in exercise, neurodegeneration, and addiction. These novel findings provide predominant evidence displaying dopamine dysregulation as previously suggested in the reward deficiency syndrome (RDS) construct. In addition, our analysis revealed important genes linked to neurodegenerative disorders (such as APOE AND TP53) having strong links to SEPP1 to exercise, aging, and even addiction through RDS. The broader implications of these data suggest a shared genetic foundation that could guide new therapeutic strategies for managing conditions related to neurodegeneration, aging, and addictive behaviors. This interconnected genetic network not only offers fresh insights into how these genes interact but also raises the possibility of developing personalized interventions that target these specific pathways to improve health outcomes.

Future studies could focus on outlining the exact role of SEPP1 and its interaction with dopamine signaling in the context of exercise, neurodegeneration, and addiction. A potential study could involve genetic manipulation to knockout SEPP1 genes in rodents, followed by measurement of dopamine receptor binding and neurodegenerative markers to assess downstream effects. Subsequent behavioral assessments could also be investigated to evaluate addiction-like behaviors, motor function, and cognitive performance. We would expect a reduction in D2R binding levels, indicating a link between SEPP1 expression and dopamine signaling. This could be accompanied by an increase in neurodegenerative markers and behavioral alterations related to motor control and cognitive function. Additionally, an increase in addictive behaviors might suggest a relationship between SEPP1 and the RDS construct.

In parallel, human studies could examine correlations between SEPP1 levels in exercised adults and dopamine transmission or neurodegenerative risk profiles. These studies might use neuroimaging techniques like PET or SPECT to measure dopamine receptor density and assess neurodegenerative markers. By examining these associations, researchers could identify specific pathways through which exercise exerts its neuroprotective effects and could better understand the potential benefits of physical activity on brain health.

## 5. Conclusions

In conclusion, our results showed increased plasma SEPP1 levels in response to increasing exercise intensity. This may be an adaptive response to the increased oxidative stress brought on by vigorous physical activity. In addition, we noticed that in our previous study, D2R binding also increased in the same rats following exercise [2,3]. This suggests that SEPP1 is associated with dopamine signaling and may be involved in the mechanism of exercise-induced D2R binding. The ability to modulate antioxidants like SEPP1 have numerous implications in health, disease, and addiction. Several environmental or epigenetic factors, including exercise intensity and duration, as well as nutritional status, may influence how exercise affects selenium metabolism and SEPP1 expression. Thus, more research is needed to determine the precise effect of exercise on selenium levels and SEPP1 in both human and animal models.

## Figures and Tables

**Table 1:** Mean [3H] spiperone (D2R) binding levels in HIIT-exercised males.

	SED ± SEM (Relative Units)	HIIT ± SEM (Relative Units)
DL Cpu	0.52 ± 0.09	0.56 ± 0.07
DM Cpu	0.38 ± 0.1	0.44 ± 0.06
VL Cpu	0.49 ± 0.1	0.55 ± 0.08
VM Cpu	0.31 ± 0.07	0.38 ± 0.07
Dorsal caudate putamen	0.29 ± 0.06	0.41 ± 0.06
Ventral caudate putamen	0.41 ± 0.1	0.59 ± 0.08
AcbC	0.22 ± 0.05	0.35 ± 0.08
AcbS	0.17 ± 0.06	0.35 ± 0.08 *
OT	0.30 ± 0.08	0.32 ± 0.09

Adapted from “High intensity interval training exercise increases dopamine D2 levels and modulates brain dopamine signaling”, by J. Tyler et al., 2023, Front Public Health 11 (https://doi.org/10.3389/fpubh.2023.1257629). Copyright 2023 by 2024 Frontiers Media S.A. accessed on 5 April 2024. [3] * *p* < 0.05 compared to SED.

**Table 2:** Mean [3H] spiperone (D2R) binding levels in MOD-exercised males.

	SED ± SEM (Relative Units)	MOD ± SEM (Relative Units)
Dorsolateral caudate putamen (DL Cpu)	0.022 ± 0.003	0.026 ± 0.002
Dorsomedial caudate putamen (DM Cpu)	0.014 ± 0.002	0.019 ± 0.002 *
Ventrolateral caudate putamen (VL Cpu)	0.02 ± 0.002	0.025 ± 0.002 *
Ventromedial caudate putamen (VM Cpu)	0.016 ± 0.002	0.023 ± 0.002 *
Nucleus accumbens core (AcbC)	0.015 ± 0.002	0.02 ± 0.002 ^#^
Nucleus accumbens shell (AcbS)	0.013 ± 0.002	0.017 ± 0.002 ^#^
Olfactory tubercle (OT)	0.007 ± 0.0008	0.009 ± 0.0004 *

Adapted from “Exercise Reduces Dopamine D1R and Increases D2R in Rats: Implications for Addiction”, by L. Robison et al., 2018, Med Sci Sports Exerc 50(8) p. 1596–1602 (https://doi.org/10.1249/MSS.0000000000001627). Copyright 2018 by the American College of Sports Medicine. accessed on 5 April 2024. [2] * *p* < 0.05 and ^#^ *p* = 0.06 compared to SED.

## Data Availability

The data presented in this study are available on request from the corresponding author.
